# APR-246 overcomes resistance to cisplatin and doxorubicin in ovarian cancer cells

**DOI:** 10.1038/cddis.2015.143

**Published:** 2015-06-18

**Authors:** N Mohell, J Alfredsson, Å Fransson, M Uustalu, S Byström, J Gullbo, A Hallberg, V J N Bykov, U Björklund, K G Wiman

**Affiliations:** 1 Aprea AB, Solna, Sweden; 2 Clinical Pharmacology, Department of Medical Sciences, Uppsala University, Uppsala, Sweden; 3 Department of Immunology, Genetics and Pathology, Uppsala University, Uppsala, Sweden; 4 Department of Medicinal Chemistry, BMC, Uppsala University, Uppsala Sweden; 5 Department of Oncology-Pathology, Cancer Center Karolinska, Karolinska Institutet, Stockholm, Sweden

## Abstract

Two main causes of platinum resistance are mutation in the tumor suppressor gene TP53 and drug-induced increase in intracellular glutathione concentration. Mutations in TP53 occur in about 50% of human tumors. APR-246 (PRIMA-1^MET^) is the first clinical-stage compound that reactivates mutant p53 and induces apoptosis. APR-246 is a prodrug that is converted to the active compound methylene quinuclidinone (MQ), a Michael acceptor that binds to cysteine residues in mutant p53 and restores its wild-type conformation. Here, we show that MQ also binds to cysteine in glutathione, thus decreasing intracellular free glutathione concentration. We also show that treatment with APR-246 completely restores the cisplatin and doxorubicin sensitivity to p53-mutant drug-resistant ovarian cancer cells. We propose that this unique ability of APR-246/MQ to bind to cysteines in both mutant p53 and glutathione has a key role in the resensitization as well as in the outstanding synergistic effects observed with APR-246 in combination with platinum compounds in ovarian cancer cell lines and primary cancer cells. However, MQ binding to cysteines in other targets, for example, thioredoxin reductase, may contribute as well. Strong synergy was also observed with the DNA-damaging drugs doxorubicin and gemcitabine, while additive effects were found with the taxane docetaxel. Our results provide a strong rationale for the ongoing clinical study with APR-246 in combination with platinum-based therapy in patients with p53-mutant recurrent high-grade serous (HGS) ovarian cancer. More than 96% of these patients carry TP53 mutations. Combined treatment with APR-246 and platinum or other DNA-damaging drugs could allow dramatically improved therapy of a wide range of therapy refractory p53 mutant tumors.

APR-246 (also called PRIMA-1^MET^) is the first compound in clinical development that reactivates mutant p53 in cancer cells by promoting its correct wild-type (wt) folding, thus triggering apoptosis.^1, 2^ The lead compound of APR-246, PRIMA-1, was originally discovered by Bykov *et al.*^3^ APR-246 showed a good safety profile in a Phase I/II clinical dose-finding study on hematological malignancies and prostate cancer and both clinical and p53-dependent biological responses were observed.^4^ A Phase Ib/II Proof of Concept study with APR-246 in combination with platinum-based therapy, in patients with recurrent p53-mutant high-grade serous (HGS) ovarian cancer, is ongoing. More than 96% of patients with HGS ovarian carcinoma carry TP53 mutations.^5^

Platinum-based drugs have an important role in the treatment of many solid tumors including ovarian cancer. Cisplatin, the first drug of this class, has had a major impact in treatment of cancer but is also associated with severe adverse effects like nephrotoxicity. This prompted the development of the less toxic analog carboplatin.^6^ The primary mechanism of action of platinum compounds is adduct formation with nucleophilic groups in tumor cell DNA. This triggers the DNA damage response pathway, in which p53 has a key role, leading to cell-cycle arrest, senescence and/or apoptosis.^7^

Patients with ovarian cancer often respond well to the first-line platinum-based chemotherapy, but the majority of the patients with advanced stage tumors relapse and eventually die of chemotherapy-refractory disease. Platinum resistance is most often associated with decreased platinum levels at the site of action (i.e., DNA) and/or failure to trigger the DNA damage response after adduct formation.^6, 7^ The underlying molecular mechanisms of resistance to platinum compounds are multifactorial, involving drug-induced increase in cellular glutathione (GSH) levels leading to enhanced efflux of platinum compounds, reduced drug uptake, increased drug inactivation and DNA adduct repair, as well as inactivation of the tumor suppressor protein p53.^7, 8, 9, 10^ Mutation in p53 is one of the main mechanisms for inhibiting propagation of the DNA damage signal to the apoptotic machinery. About 50% of all tumors carry mutant p53 (see p53.free.fr, 2015) and cancer cells with defects in p53 are in general more resistant to conventional chemotherapy. In many tumors, including ovarian cancer, p53 mutations are correlated to shortened time to progression and decreased patient survival time.^11, 12^ Thus, restoration of wt function of p53 is a promising strategy for cancer therapy.^13, 14^

Here, we describe a new aspect of therapeutic activity of APR-246. APR-246 not only reactivates p53 but also decreases intracellular glutathione levels in a dose-dependent manner. Moreover, APR-246 completely restored cisplatin and doxorubicin sensitivity to mutant p53-carrying resistant ovarian cancer cells. Our results may open possibilities for greatly improved treatment of a wide range of platinum-resistant tumors.

## Results

### APR-246 resensitizes cisplatin-resistant ovarian cancer cells to cisplatin

**Figure 1:**
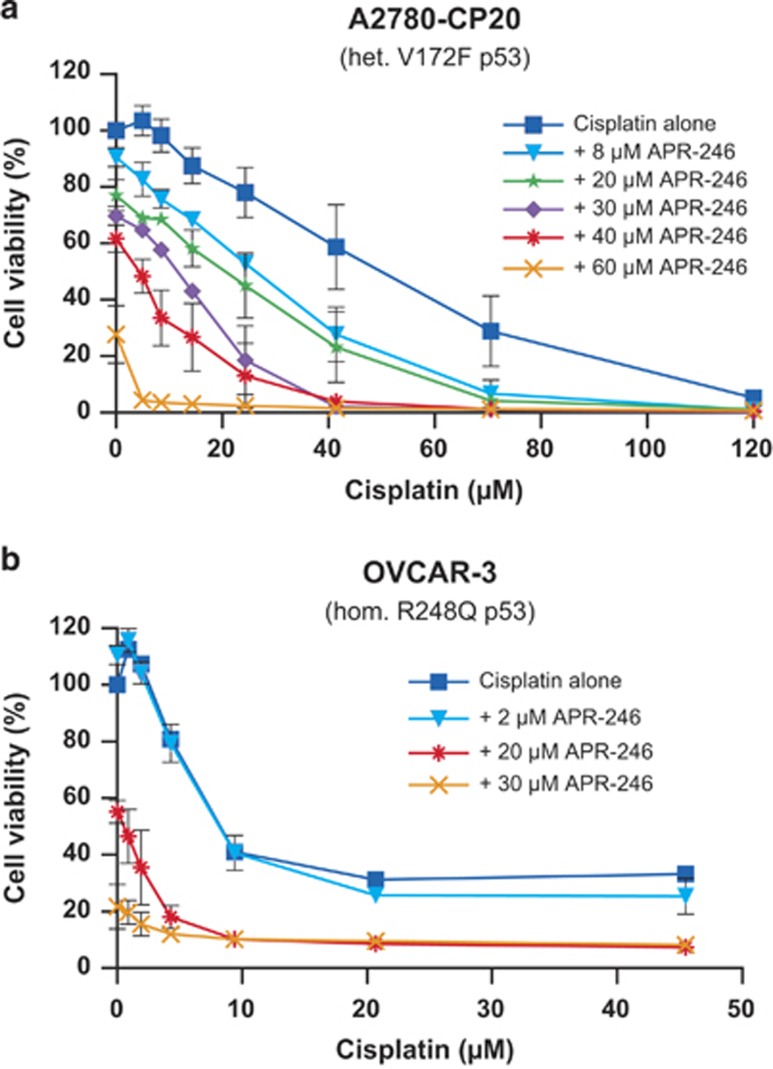
(**a** and **b**) APR-246 resensitized the cisplatin-resistant ovarian cancer cell lines A2780-CP20 and OVCAR-3 to cisplatin. The FMCA was used for measurement of cell viability. The results shown are mean±S.E.M. (*n*⩾2)

We first investigated whether APR-246 could resensitize the p53-mutant cisplatin-resistant A2780-CP20 and OVCAR-3 ovarian cancer cells to cisplatin using cell viability assay. The A2780-CP20 ovarian adenocarcinoma cell line carries a V172F mutation and was developed by chronic *in vitro* exposure of the parental A2780 cells to increasing concentrations of cisplatin.^15^ The OVCAR-3 cells with hotspot p53 mutation (R248Q) were established from malignant ascites of a patient with progressive adenocarcinoma of the ovary.^16^ The patient had been treated with cisplatin, doxorubicin and cyclophosphamide and was clinically resistant to cisplatin and doxorubicin.^16^ Dose-response experiments with cisplatin alone and in combination with various concentrations of APR-246 were performed. As shown in Figure 1a, APR-246 resensitized A2780-CP20 cells to cisplatin in a dose-dependent manner. The IC_50_ value of cisplatin (with the partial effect contribution from APR-246 subtracted) decreased 18-fold from 52±11 to 3.2±0.8 *μ*M (mean±S.E.M.; *P*<0.05; *t*-test), which is slightly lower than the IC_50_ value of cisplatin in A2780 cells (3.7±0.67 *μ*M). Thus, APR-246, at clinically relevant concentrations, completely restored the sensitivity of the ovarian cancer cells to cisplatin.

APR-246 also resensitized OVCAR-3 cancer cells to cisplatin (Figure 1b). The IC_50_ value of cisplatin decreased 3.2-fold, from 8.3±0.2 *μ*M to 2.6±0.9 *μ*M (mean±S.E.M.; *n*=2) in the presence of 20 *μ*M APR-246. Interestingly, in addition to increasing the sensitivity of the cells to cisplatin (i.e., decreasing the IC_50_ value), APR-246 appeared to increase the efficacy of cisplatin by reducing the survival index plateau at higher concentration from 30 to 5%.

### Strong synergistic effects of APR-246 and platinum compounds in drug-resistant ovarian cancer cells

**Figure 2:**
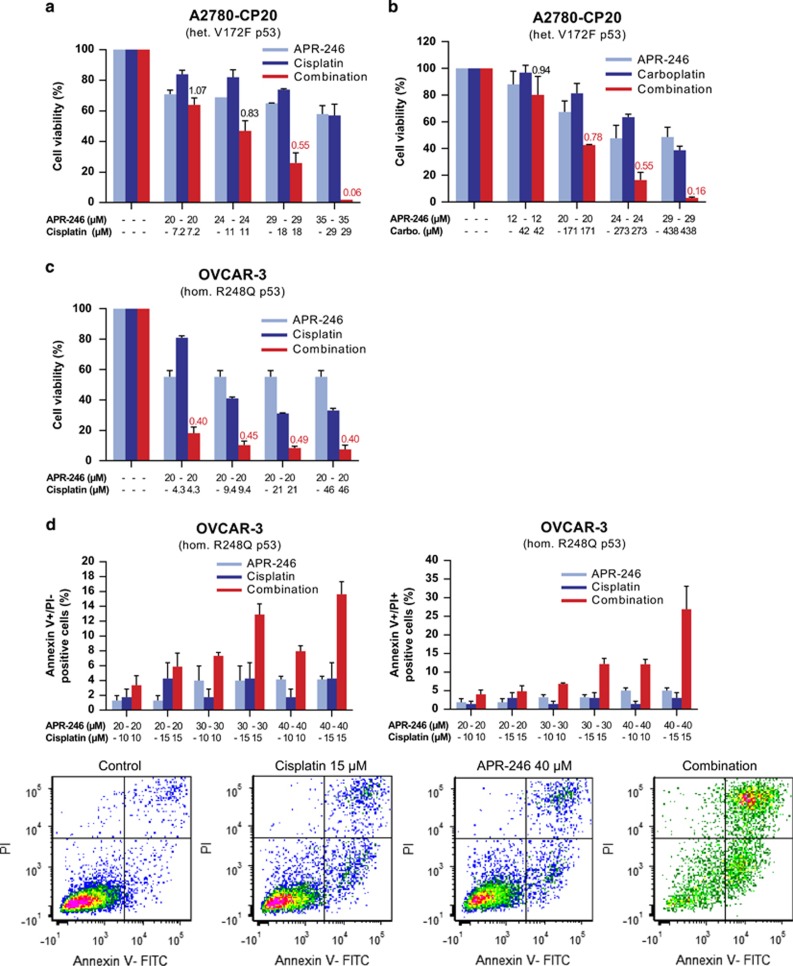
Combination studies with APR-246 and platinum compounds in drug-resistant ovarian cancer cells. (**a**–**c**) Synergistic effects of APR-246 and platinum compounds in ovarian cancer cell lines A2780-CP20 and OVCAR-3. The FMCA (in **a**–**c**) was used for measurement of cell viability. Additive model was used for analysis of combination effects. CI values are presented above the bars. CI<0.8 indicates synergistic, <0.5 strong synergistic, and <0.3 outstanding synergistic effect. CI values <0.8 are marked in red. (**d**) Synergistic effects of APR-246 and cisplatin on apoptosis in OVCAR-3 cells. Apoptosis was determined using Annexin V apoptosis detection kit and analyzed by flow cytometry. Factorial ANOVA indicated statistically significant synergistic effect between cisplatin and APR-246 in the induction of both early and late apoptosis (*P*<0.01). The results shown are mean±S.E.M. (*n*≥2)

We then investigated whether APR-246 acts synergistically with cisplatin or carboplatin in cisplatin-resistant ovarian cancer cell lines. We found outstanding synergy (combination index (CI)<0.3) with cisplatin (Figure 2a) or carboplatin (Figure 2b) in A2780-CP20 cancer cells. Outstanding synergy was also found in the mutant p53-carrying (Y126C and R337C) cisplatin-resistant ovarian cancer cell line IGROV-1/CDDP (Supplementary Figure S1a and b), which has been established by exposure of the parental wt p53-carrying IGROV-1 cells to cisplatin.^17^ The IGROV-1 cell line was established from an untreated ovarian cancer patient.^18^ Moreover, we observed strong synergistic effects with APR-246 and cisplatin in OVCAR-3 cells (Figure 2c). Furthermore, strong synergy (CI<0.5) was found in the wt p53-carrying parental A2780 cell line, which was established from an untreated cancer patient^19^ and outstanding synergy in the cisplatin-resistant A2780cis subline harboring wt p53 (Supplementary Figure S1c and d, respectively).^20^ The results from these studies are summarized in Table 1a. Finally, we investigated the effects of APR-246, cisplatin and their combination on apoptosis and reactive oxygen species (ROS) in OVCAR-3 cells. The synergistic response was evident based on emerging fractions of Annexin V+/PI− (early apoptotic) and Annexin V+/PI+ (late apoptotic/necrotic) cells (Figure 2d), as well as based on ROS induction (Supplementary Figure S2).

### Cross-resistance

Treatment with cisplatin results not only in primary resistance but also in cross-resistance to other platinum compounds and classical alkylating agents, as well as anthracyclines including doxorubicin.^21^ We performed dose-response experiments with cisplatin, carboplatin, doxorubicin and APR-246 in the A2780 line and its drug-resistant sublines A2780cis, A2780-CP20 and A2780ADR. The A2780ADR cells have wt p53 and have been developed by exposure of the A2780 cells to doxorubicin.^22^ The results are summarized in Table 1b. The IC_50_ values of cisplatin in A2780, A2780cis and A2780-CP20 were 3.7, 18 and 40 *μ*M, respectively. Thus, the IC_50_ value was increased 4.8-fold in the A2780cis cells carrying wt p53, and 11-fold in the mutant p53-carrying A2780-CP20 cells. The cisplatin-resistant sublines were cross-resistant to carboplatin and doxorubicin. The doxorubicin-resistant A2780ADR cells showed 18-fold resistance to doxorubicin and were cross-resistant to cisplatin and carboplatin. The IC_50_ value of APR-246 was less affected and was increased 1.6-fold in A2780-CP20 cells, whereas there was a 2-fold decrease in A2780ADR cells.

### Synergistic effects of APR-246 and cisplatin in lung cancer cell lines

We also tested the effect of APR-246 in combination with cisplatin in small cell lung cancer (SCLC) and non-small cell lung cancer (NSCLC) cell lines with various p53 mutations. Strong synergistic effects with APR-246 and cisplatin were observed in all cancer cell lines with hotspot p53 mutations (R248Q, R248W, R273H and G245C). These cells expressed high levels of mutant p53 (Table 1a; Supplementary Figure S3). Mutant p53 often accumulates at high levels in cancer cells, which is believed to contribute to the strong apoptotic response upon APR-246 treatment.^3^ Strong synergy was also seen in the SCLC cell line NCI-H378 with the Y163C p53 mutation that is not considered as a hotspot mutation but still occurs frequently in tumors. These cells expressed a lower level of p53 than the cells with hotspot p53 mutations (Supplementary Figure S3). Synergistic or strong synergistic effects were also observed in the lung cancer cell line NCI-H1417 with frameshift mutation in TP53 and no expression of full-length p53 (Table 1a; Supplementary Figure S3).

### Synergistic effects with APR-246 and cisplatin in primary ovarian cancer cells

Strong synergistic effects were observed in primary tumor cells from all five ovarian cancer patients included in the study (Table 1c). DNA sequencing revealed that four of them had TP53 mutations. One of the mutations was the relatively frequently occurring Y220C mutation, whereas three patients had frameshift or nonsense mutations (Table 1c).

### *In vivo* antitumor effect of APR-246 in combination with cisplatin

**Figure 3:**
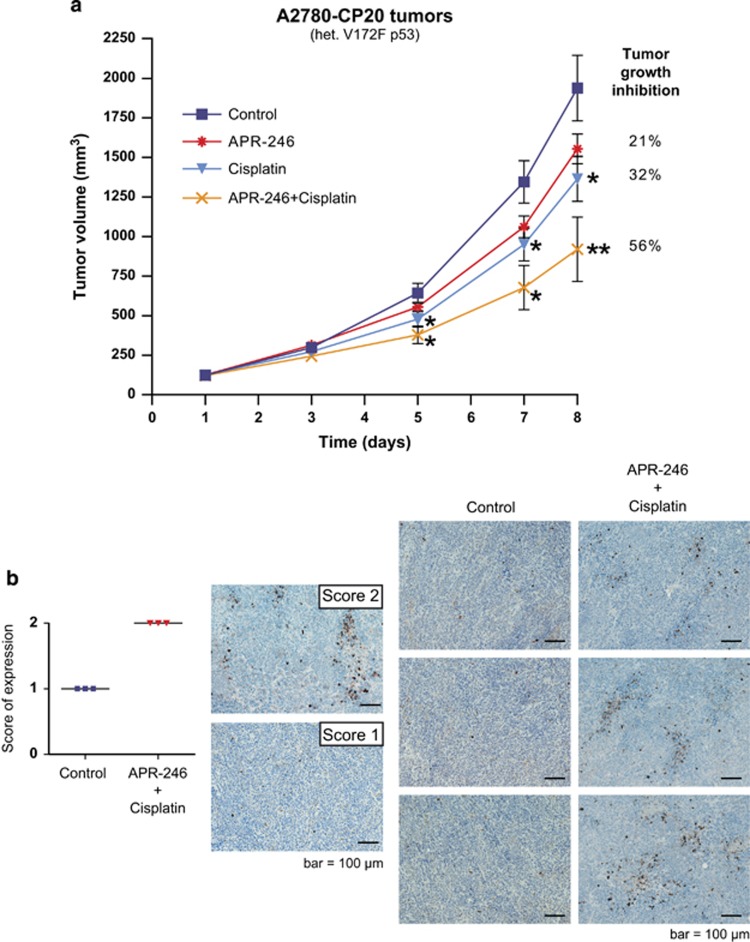
*In vivo* effects of APR-246 in combination with cisplatin on p53-mutant ovarian A2780-CP20 tumors in mice. (**a**) Inhibition of tumor growth. APR-246 was administered as 2 h continuous i.v. infusion (400 mg/kg/day, treatment days 1–7). Cisplatin was administered as i.v. bolus injection (4 mg/kg/day, treatment days 2 and 6). The results are shown as mean±S.E.M. (*n*=10). Mann–Whitney U-test was used for statistical analysis of differences in tumor growth between treatment groups compared with control. *P<0.05; **P<0.01. (**b**) Activation of caspase-3. The treatment group had the same treatment schedule as the combination group in the *in vivo* efficacy study shown in (**a**) (3 mice per group). Tumor sections were immunohistochemically stained for active Caspase-3. Left panel: pictures are representative examples of evaluation scores; Score 1 (+): minimal amount of positive cells; Score 2 (++): moderate amount of positive cells. Right panel: Representative picture of each tumor analyzed

The antitumor effect of APR-246 in combination with cisplatin in mice bearing the aggressively growing A2780-CP20 tumor xenografts was examined. As shown in Figure 3a, single treatment with APR-246 and cisplatin inhibited tumor growth by 21 and 32%, respectively, while the combination resulted in 56% inhibition of tumor growth, indicating at least an additive effect. It should be noted that these doses were chosen to allow detection of a combination effect rather than to achieve maximal anticancer effect. Toxicity was evaluated on the basis of body weight reduction and observation of clinical signs of adverse effects. APR-246 was well tolerated and the general condition of the animals was good throughout the study. In the combination treatment group, the maximal body weight reduction was 10% and the mice recovered weight promptly after the treatment.

Using the same *in vivo* cancer model and treatment schedule, we examined the effect of combination treatment with APR-246 and cisplatin on activation of effector caspase-3, a marker of apoptosis. Analysis by immunohistochemistry showed an increase in active caspase-3-positive cells in all tumors (Figure 3b).

### MQ is the active compound

**Figure 4:**
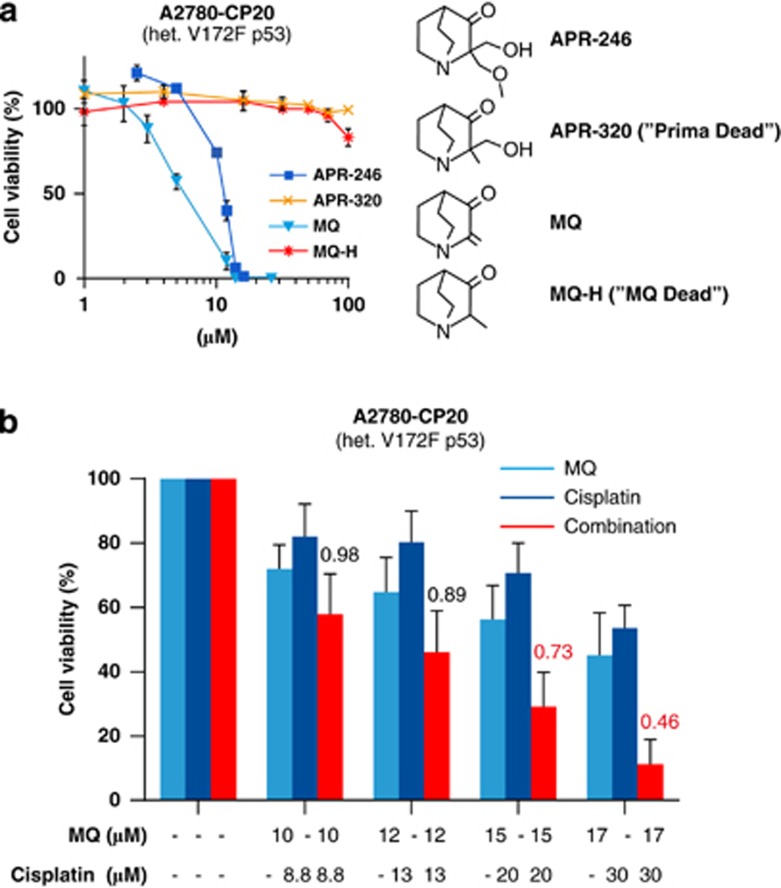
MQ is the active moiety of APR-246. (**a**) Effect of APR-246, MQ, APR-320 and MQ-H on viability of ovarian cancer A2780-CP20 cells. The WST-1 assay was used for measurement of cell viability. The results are shown as mean±S.E.M. (*n*=2). (**b**) Synergistic effects of MQ and cisplatin on cell viability of A2780-CP20 cells. FMCA was used for measurement of cell viability and Additive model for analysis of results. CI values are presented above the bars. CI<0.8 indicates synergistic and <0.5 strong synergistic effects. CI values <0.8 are marked in red. Results are shown as mean±S.E.M. (*n*=3)

APR-246 is a prodrug that is converted to MQ (2-methylenequinuclidin-3-one) and available evidence strongly suggests that MQ is the active compound responsible for the anticancer effects of APR-246.^1^ To further investigate this, we compared the effect of MQ and APR-246 on cell viability of A2780-CP20 ovarian cancer cells. Both APR-246 and MQ reduced the A2780-CP20 cell viability in a dose-dependent manner (Figure 4a). MQ was 2.3-fold more potent than APR-246, with IC_50_ values of 4.8±0.4 *μ*M and 11±0.1 *μ*M, respectively (mean±S.E.M.; *P*<0.05; *t*-test). In contrast, neither APR-320, a structural analog of APR-246 that cannot be converted to MQ, nor the MQ analog MQ-H that lacks Michael acceptor activity, had any effect on cell viability. Moreover, as shown in Figure 4b, MQ had strong synergistic effect with cisplatin in A2780-CP20 cells. These results are consistent with our previous results^1^ and provide further support for MQ being the active compound.

### APR-246 decreases intracellular glutathione levels

Many studies have shown that glutathione, which has an important role in maintaining the cellular oxidative balance, is involved in resistance to DNA-damaging drugs including platinum compounds and classical alkylating agents.^7, 15, 21, 23, 24, 25^ Intracellular glutathione exists in a balance between the reduced form (GSH), which constitutes the major fraction and is present at mM levels, and the oxidized form (GSSG). The drug-induced increase in intracellular glutathione concentration leads to, for example, increased efflux of cisplatin through ATP-binding cassette (ABC) transport pumps. A good correlation between intracellular glutathione levels and the degree of resistance to cisplatin has previously been shown in a panel of ovarian cancer cell lines, including the cell lines investigated in our study.^15^

**Figure 5:**
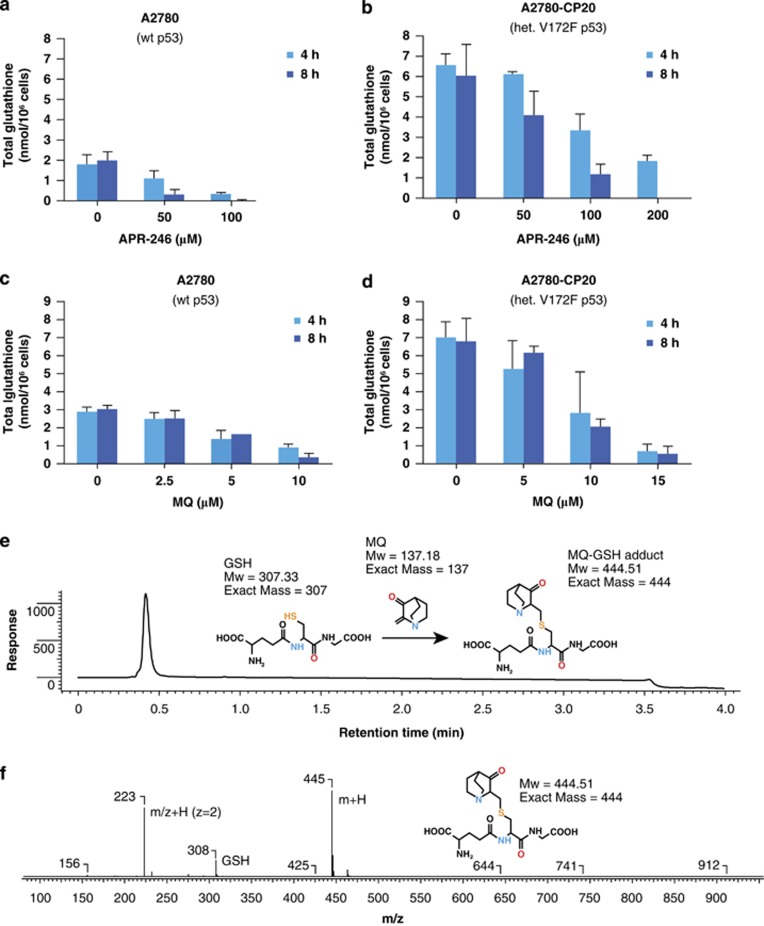
APR-246 and MQ reduce glutathione levels in ovarian cancer cells. (**a**–**d**) Effects of APR-246 and MQ on intracellular glutathione levels in A2780 and in A2780-CP20 ovarian cancer cells. Total GSH levels (GSH+2 × GSSG) were measured using Cayman Glutathione Assay kit. The results are shown as mean±S.E.M. (*n*=2). (**e** and **f**) MQ forms a Michael adduct with glutathione. (**e**) Liquid chromatography trace of the reaction mixture after addition of excess glutathione (GSH) shows total consumption of MQ. Product and GSH co-eluates under the conditions used. (**f**) Mass spectrometry (MS) of the retention time 0.42 peak shows the typical MS pattern of the GS-MQ adduct (m/z: 445 +223). MS-peak of GSH (in excess): m/z: 308 [m+H]. The typical MS pattern of the GS-MQ adduct (m/z: 445 and 223) and the slight excess of added GSH to the reaction mixture is shown by the MS peak m/z=308 [m+H]. The results shown in (**e**–**f**) are representative of three independent experiments

Figure 5 shows that the glutathione levels were about 3-fold higher in the cisplatin-resistant A2780-CP20 cells than in the A2780 cells, in agreement with the previous results.^15^ In both cell lines, APR-246 (Figures 5a and b) and MQ (Figures 5c and d) decreased glutathione in a dose-dependent manner, resulting in depletion of free glutathione at higher concentrations. Cisplatin alone did not have any significant effect on glutathione levels, while combination treatment with APR-246 and cisplatin resulted in more than additive effects (Supplementary Figure S4). Notably, APR-246 (50 *μ*M, 8 h) decreased glutathione levels equally (i.e., 2 nmol glutathione/10^6^ cells) in wild-type and p53-mutant cell lines (Figures 5a and b), suggesting that reactivation of p53 as such did not have any additional effect on glutathione levels.

### MQ binds to glutathione

We then tested whether MQ reacts with glutathione. As shown in Figures 5e and f, MQ reacts rapidly with glutathione to form a Michael adduct. No reverse reaction was observed under these conditions. Analysis by HPLC-MS (Figure 5e) revealed a peak of the glutathione-MQ (GS-MQ) adduct at retention time 0.42, which was verified by the mass spectrometry (MS) (Figure 5f). No LC-MS signals corresponding to remaining MQ could be detected, indicating that the slight excess of GSH quickly consumed MQ.

### Combination effects of APR-246 with doxorubicin

**Figure 6:**
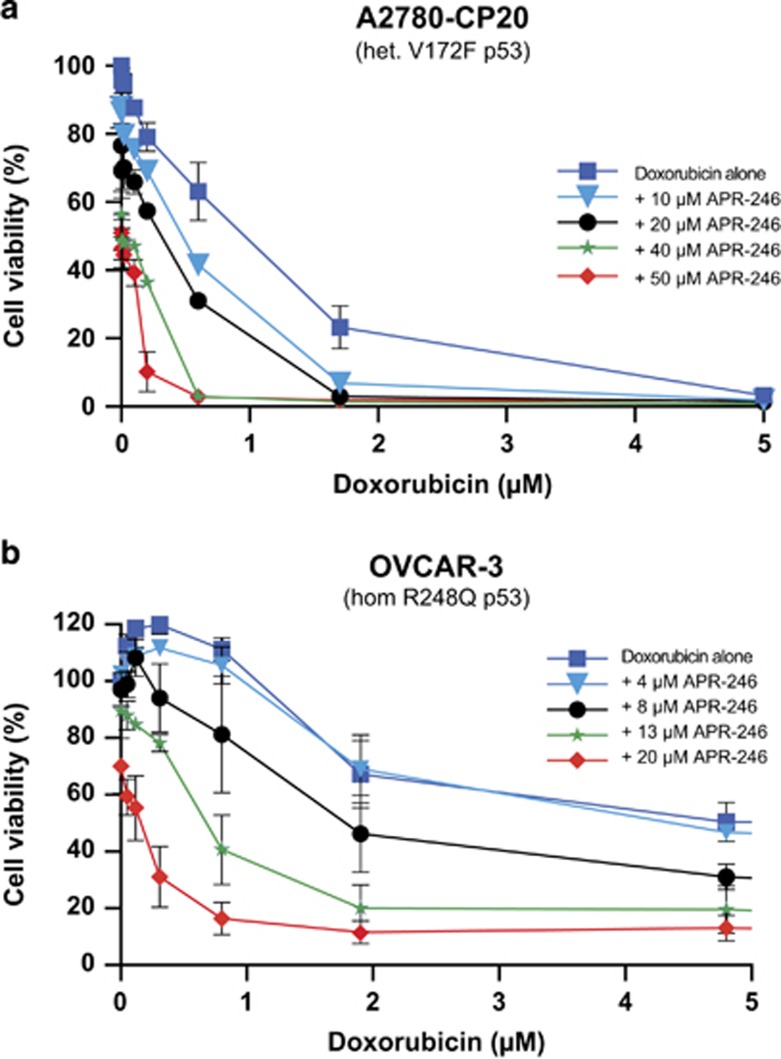
APR-246 resensitized the A2780-CP20 (**a**) and OVCAR-3 cells (**b**) to doxorubicin. The FMCA was used for measurement of cell viability. The values are mean±S.E.M. (*n*=2)

The main mechanisms of actions of anthracyclines, including doxorubicin, are inhibition of DNA and RNA synthesis by intercalation between base pairs of the DNA/RNA strands, and interference with the topoisomerase II enzyme, leading to double-strand breaks.^26^ This results in a DNA damage response, including activation of the p53 pathway leading to apoptosis. Although it has been reported that mutation in p53 and the p53 pathway can cause resistance to anthracyclines,^27^ the significance of p53 status for sensitivity to anthracyclines is less well documented than its role in the response to platinum compounds. We found that APR-246 completely restored the sensitivity of the A2780-CP20 cells to doxorubicin; the IC_50_ value of doxorubicin decreased 8-fold from 0.95±0.11 *μ*M to 0.12±0.01 *μ*M (mean±S.E.M.; *n*=2) (Figure 6a), which is equal to the IC_50_ value in the parental cells (0.12±0.05 *μ*M). APR-246 also resensitized OVCAR-3 cells to doxorubicin (Figure 6b). Similarly to the experiment with cisplatin (Figure 1b), APR-246 appeared to increase the efficacy of doxorubicin by reducing the survival index plateau. Moreover, we observed outstanding synergy with APR-246 in combination with doxorubicin in doxorubicin-resistant A2780ADR ovarian carcinoma cells (Supplementary Figure S1f). In the parental ovarian cancer A2780 cells, additive and synergistic effects with APR-246 and doxorubicin were observed (Supplementary Figure S1e).

### Synergy with gemcitabine but not with docetaxel

The DNA-damaging drug gemcitabine is a nucleoside analog that replaces cytidine during DNA replication, leading to tumor growth arrest and eventually apoptosis. Strong synergistic effect with APR-246 and gemcitabine was observed in the A2780-CP20 cells (Supplementary Figure S1g). The mechanisms underlying the synergistic effect with APR-246 and gemcitabine have not been further investigated, but it has been reported that p53 and glutathione are involved also in resistance development for gemcitabine.^28^

Notably, we did not observe any synergy between APR-246 and the taxane docetaxel in A2780-CP20 cells (Supplementary Figure S1h). Taxanes act by disrupting the function of the microtubules. Thus, they have a clearly different mechanism of action compared with the DNA-damaging drugs carboplatin, cisplatin, doxorubicin and gemcitabine. No clear role of p53 in the mechanism of action or resistance development to taxanes has been shown.^29, 30^

## Discussion

Platinum compounds are among the most effective anticancer drugs known, and have been used as a first-line treatment of several solid tumors, including ovarian cancer. Their main mode of action is interaction with DNA to form DNA adducts, which leads to a DNA damage response involving activation of p53-dependent apoptosis. However, repeated treatment with platinum drugs rapidly results in attenuation of the DNA damage response and resistance. Two of the main causes of resistance are p53 mutations and drug-induced increase in intracellular glutathione concentration.

**Figure 7:**
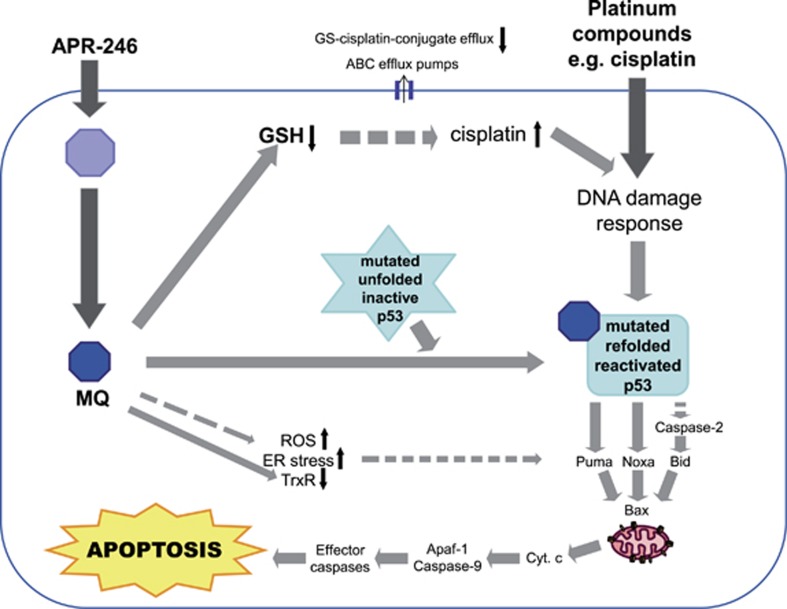
Schematic drawing of the mechanism of action of APR-246 in combination with platinum compounds. Filled arrows indicate direct effect and dashed arrows indicate non-direct effect. MQ can also inhibit glutathione synthesis by binding to free cysteines

The mode of action of APR-246 as a mutant p53-targeting anticancer compound is well documented.^1, 2, 31, 32^ APR-246 reactivates mutant p53 and induces expression of pro-apoptotic p53 target genes including Puma, Noxa and Bax, followed by activation of the mitochondrial apoptosis pathway.^32^ APR-246 can also trigger apoptosis in a p53-independent manner by inducing ROS and endoplasmic reticulum (ER) stress^1, 2^ and by inhibiting thioredoxin reductase 1 (TrxR1) (Figure 7).^33^ Recently, Tessoulin *et al.*^34^ reported that APR-246 induced cell death in myeloma cells independently of p53 status by impairing the GSH/ROS balance.

APR-246 is a prodrug that is converted to the active compound MQ, a Michael acceptor and consequently a soft electrophile that reacts reversibly and preferentially with soft nucleophiles such as thiols in cysteines in p53. The p53 core domain has 10 cysteine residues to which MQ can potentially bind and stabilize p53 wild-type conformation.^1^ Due to favorable molecular orbital interactions, high selectivity is achieved in comparison with classical hard electrophiles as the alkylating agents frequently used in cancer therapy. Certain other compounds that were identified based on their ability to target mutant p53-expressing cells are also Michael acceptors that can form adducts with thiol groups.^35, 36^ One of these, MIRA-1, had promising properties *in vitro* but is toxic *in vivo,* probably because it binds to multiple protein targets extracellularly, resulting in toxicity.^36^ Thus, the optimal mutant p53-reactivating compound may be a prodrug such as APR-246 that is converted to the active compound intracellularly.

Our results show that MQ, in addition to binding to cysteines in p53, also binds to the cysteine in glutathione, a tripeptide formed by glutamic acid, cysteine and glycine, decreasing intracellular free glutathione levels in ovarian cancer cells. It is possible that MQ also binds to free cysteine and thereby inhibits glutathione synthesis. Moreover, APR-246/MQ induces formation of ROS in tumor cells,^1^ which can lead to a further decrease in intracellular glutathione concentration. These multiple effects of APR-246/MQ presumably explain that APR-246, at clinically relevant concentrations, can deplete intracellular glutathione in ovarian cancer cells (Figure 5).

In addition to mutant p53, MQ can bind to unfolded inactive wt p53 and promote its correct folding.^1^ It is conceivable that p53 protein unfolding leads to exposure of cysteine residues that can be modified. Indeed, APR-246 has been shown to activate wt p53 in melanoma cells in which p53 is inactivated by integrin αv-mediated signalling.^37^ The proposed mechanisms of action can also explain the synergistic effects of APR-246 and platinum compounds observed in cisplatin-resistant cells that carry wt p53. In these cells, the synergy could be mainly due to decreased glutathione levels, although stabilization of wt p53 by MQ may contribute as well. In cancer cells that carry homozygous frame shift or nonsense mutations and therefore do not express full-length p53, the synergy could also be due to APR-246 effects on glutathione. Further studies are ongoing to further explore the molecular mechanism underlying the synergistic effects in cancer cells with various p53 status.

Most tumor-associated p53 mutations are missense mutations located in the DNA-binding core domain of p53. The most frequent p53 mutations, so-called hotspot mutations, affect amino-acid positions R175, G245, R248, R249, R273 and R282. In many cases, mutant p53 proteins have prolonged half-life and accumulate within cancer cells.^38^ Many frequent mutations may also confer so-called gain-of-function activities to mutant p53.^38^ We observed strong synergy with APR-246 and cisplatin in all cancer cells harboring homozygous hotspot mutations (Table 1a). This is consistent with our previous studies showing that APR-246 can reactivate a wide range of mutant p53 proteins,^1, 3, 39^ and is also consistent with data from us and others showing that PRIMA-1 and APR-246 can synergize with chemotherapeutic drugs including cisplatin and doxorubicin.^31, 40, 41^ Here, we have explored combination treatment with APR-246 and DNA-damaging drugs in a more systematic and quantitative manner, using for example higher concentrations of APR-246 that result in stronger synergies. We have also used a broader range of cancer cell lines carrying different mutant forms of p53, with the aim of understanding the molecular mechanisms underlying the synergistic effects. Many of the cell lines examined in this study express high levels of mutant p53, which may contribute to the strong apoptosis-inducing effect of APR-246. Since p53 is a tetramer of four p53 monomers, heterozygous mutations may compromise the function despite the presence of a wt allele.^42^

Resistance to chemotherapy is a major obstacle to clinical use of most chemotherapeutic agents, and numerous attempts to restore the chemosensitivity have been made. Much effort has focused on the ABC-drug transporters that have been shown to be overexpressed in many cancer cell lines as well as clinical samples, and cause reduced drug concentrations in cancer cells.^43^ Co-administration of efflux pump inhibitors increases intracellular drug concentrations *in vitro*. However, clinical trials testing this paradigm have mostly failed,^44^ presumably due to poor selectivity of the inhibitors resulting in intolerable side effects.^45^ The glutathione system has also received attention, and based on encouraging results from animal models several clinical trials with buthionine sulfoximine (BSO), an inhibitor of glutathione synthesis, have been performed.^46^ While BSO alone produced minimal toxic effects, combinations with melphalan occasionally resulted in severe myelosuppression.^47^

Based on the results presented here we propose a unique mechanism of action of APR-246, in combination with platinum compounds, which distinguishes it from other anticancer drugs as well as other drugs that modulate platinum resistance (Figure 7). APR-246 is converted to MQ that binds to cysteine residues in mutant p53 and promotes refolding of the core domain. This provides a strong pro-apoptotic signal by itself, and also enhances the apoptotic response to platinum drugs that require functional p53 to exert their effect. In addition, MQ binds to the cysteine residue in glutathione and decreases intracellular glutathione concentration resulting in potentiation of the effect of platinum compounds. The synergistic effects observed with APR-246 and the DNA-damaging drugs doxorubicin or gemcitabine could at least in part be explained by the same mechanisms. This dual/multiple mechanism of action of APR-246 may provide a novel paradigm for overcoming platinum resistance in cancer therapy. Our results provide a strong rationale for the ongoing study with APR-246 in combination with carboplatin and pegylated liposomal doxorubicin in patients with recurrent ovarian cancer expressing mutant p53, and suggest that combination treatment with APR-246 and platinum or other DNA-damaging drugs could allow dramatically improved therapy of a wide range of therapy-refractory human tumors carrying mutant p53.

## Materials and Methods

### Test substances

APR-246 (2-hydroxymethyl-2-methoxymethyl-1-azabicyclo [2,2,2] octan-3-one) and MQ (2-methylenequinuclidin-3-one) were from Aprea (Solna, Sweden). Cisplatin (Ebewe or Hospira), carboplatin (Hospira), docetaxel (Actavis) and doxorubicin (Teva) were purchased from the Pharmacy at Akademiska sjukhuset, Uppsala, Sweden. Cisplatin was also purchased from Sigma (St. Louis, MO, USA). Gemcitabine was from LC Laboratories (Woburn, MA, USA), and glutathione from Sigma-Aldrich (Steinheim, Germany).

### Cell lines and cell culturing

Information about cell lines, including authentication and culture conditions, are described in Supplementary Table S1.

### Primary cells

Human cancer tissue samples were obtained from Capital Biosciences (Rockville, MD, USA) and Genscript (Piscataway, NJ, USA). They had been enzymatically dispersed and filtered through 100–150 *μ*m filters, and the tumor cells were viable frozen and shipped to Aprea. The quality of the tumor cells was visually judged by a cytopathologist. According to quality criteria at least 70% of cells should be cancer cells and the cell viability should be at least 70%. Tissues were collected using Informed Consent, and the procedures were supported by ethics committees and were in accordance with the principles of the Declaration of Helsinki.

### Analysis of TP53 gene status

All cell lines and primary samples were analyzed for *TP53* gene status (exons 2–11) by PCR amplification followed by single-strand conformation analysis (SSCA) according to the original protocol^48^ and samples displaying gel mobility shifts were sequenced to confirm the nucleotide change. The analysis of *TP53* gene status was performed at Department of Clinical and Experimental Medicine, Linköping University.

### Western blotting

Cells were analyzed by western blotting. Primary antibodies were anti-p53 antibody (#9282, Cell Signaling, Danvers, MA, USA), anti-p53 antibody (#FL-393, Santa Cruz, Dallas, TX, USA) and anti-GAPDH antibody (#5632-1, clone EPR6256, Epitomics, Abcam, Cambridge, UK). Secondary antibodies were goat anti-mouse HRP-conjugated antibody (#P 0447, Dako, Glostrup, Denmark), goat anti-rabbit HRP-conjugated antibody (#P 044801-2, Dako). The experiments were performed at the Department of Clinical and Experimental Medicine, Linköping University.

### Cell viability assays

Cell viability assays used were FMCA, WST-1, Cell Titer-Glo and MTS assay. In all, 3000–12 000 cells/well in 96-well plates were incubated for 72 h, at 37 °C and with 5% CO_2_ before analysis. The FMCA and WST-1 assays were performed by Aprea, the Cell Titer-Glo assay by Accelera and the MTS assay by Oncodesign.

### Annexin V/PI assay

OVCAR-3 cells were plated at a density of 75 000 cells per well in 3 ml of medium in 12-well plates. Next day, 2.5 ml medium was removed and cells were treated with cisplatin or APR-246 or in combination for 20 h. Next day, cells were harvested by trypsinization, washed twice and cells were stained with Annexin V and propidium iodine (PI) (FITC Annexin V apoptosis detection kit I, BD Biosciences, Stockholm, Sweden). After staining, the samples were analyzed by LSRII flow cytometer (BD Biosciences).

### Measurement of intracellular ROS generation

The procedure was done exactly as described in the experiment above until trypsinization, then cells were stained with 2′7′-dichlorofluorescin diacetate (DCF-DA) (Sigma) (5 μg/ml) in PBS with Ca^2+^/Mg^2+^ for 30 min at 37 °C. After staining the samples were analyzed by LSRII flow cytometer (BD Biosciences).

### Analysis of results from combination studies

For investigating possible additive and synergistic effects when using combinations of drugs, the data were analyzed with the Additive model.^49, 50^ Some of the results were also analyzed according to the Chou-Talalay model.^51^ However, this model was less suitable for combination studies with APR-246 due to the considerably steeper dose-response curve with APR-246 than with platinum compounds and doxorubicin. In those studies where it could be used, similar results with Additive and Chou-Talalay model were obtained (results not shown). Factorial ANOVA model was used to evaluate the interaction between cisplatin and APR-246 in the apoptosis and ROS studies in OVCAR-3 cells.

### *In vivo* xenograft efficacy study

A2780-CP20 cells were injected s.c. into the left flank of female CD-1 Nu/Nu mice (5 × 10^6^ cells/mice) (Charles River, Italy). The mice were 5 weeks old and weighed 18–26 g. Treatment started when the mean tumor volume was ~120 mm^3^. APR-246 (in PBS) was administered as 2 h continuous i.v. infusion/day on treatment days and cisplatin (in water) as i.v. bolus injection immediately before APR-246 infusion on treatment days 2 and 6. Treatment volumes were 10 ml/kg. Tumors were measured with a caliper. Toxicity was evaluated on the basis of the body weight reduction. The mice were observed for clinical signs daily. The *in vivo* xenograft efficacy study was performed by Accelera. All procedures adopted for housing and handling of animals were in strict compliance with Italian and European guidelines for laboratory animal welfare.

### Evaluation of active caspase-3 in tumors

Inoculation of tumor cells, strain and age of mice, handling procedures and ethical permissions were the same as in the *in vivo* efficacy study. Mouse weights were 20–24 g. Mice were randomized into two groups of three mice. To have homogeneity of tumor size, the mice were killed when the mean tumor size in each group was 0.68±0.25 cm^3^. At the scheduled time points, tumors were excised (in the treatment group 4 h after the end of the last infusion), formalin fixed, paraffin embedded, stained using primary monoclonal anti-active Caspase-3 antibody (Cell Signaling), and visualized by DAKO EnVision+Rabbit Polymer System and a Zeiss microscope (Axioscope-2 plus, Carl Zeiss, Göttingen, Germany). The stained tumor sections were examined in blind by two independent observers. The study was performed by Accelera.

### Analysis of MQ binding to glutathione

GSH (97.7 *μ*mol) was added to a solution of MQ (73.0 *μ*mol) in 1 ml of de-ionized water containing NaHCO_3_ (~20 mg) resulting in a pH of ~7. The mixture was stirred for 5 min at room temperature and analyzed by tandem HPLC-MS on an Agilent Series 1100 system using an ACE C8 (3 *μ*m, 3.0 × 50 mm) column and a mobile phase flow rate of 1 ml/min with a gradient of 10–97%, or 30–80% CH_3_CN in 10 mM NH_4_HCO_3_ buffer over 3 min. UV detection was performed at 220 nm. Electrospray mass spectrometry (ES-MS) was performed using an Agilent 1100 Series Liquid Chromatograph/Mass Selective Detector (MSD) to obtain the pseudo molecular [M+H]^+^ ion of the target molecules.

### Glutathione assay

In all, 30 000 cells/cm^2^ were seeded in 75 cm^2^ flasks (2.25 × 10^6^ cells/flask in 15 ml of medium) and incubated for 24 h before treatment with APR-246 for 4 and 8 h. Cells were harvested and counted, and intracellular total glutathione levels (glutathione [GSH] and glutathione disulfide [GSSG]) were measured using a Glutathione Assay Kit (Cayman Chemical Company, Ann Arbor, MI, USA). This kit contains glutathione reductase that converts GSSG to GSH. Cell diameters were measured using a cell Coulter counter, assuming spherical cells. No significant effect on cell viability was observed at the indicated concentrations of APR-246 and MQ. These experiments were performed by Accelera. For more detailed information about the Materials and Methods, see Supplementary Methods, additional Information.

## Figures and Tables

**Table 1a:** Results from combination studies with APR-246 and cisplatin in ovarian and lung cancer cell lines

**Cancer cell lines**	**Cancer type**	**p53 status**	**p53 protein expression**	**Combination APR-246 and cisplatin**
*Ovarian*				
OVCAR-3	Ovarian	R248Q (hom.)	+++	SS
A2780-CP20	Ovarian	V172F (het.)	+	SS
IGROV-1/CDDP	Ovarian	Y126C (het.), R337C (het.)	+	S/SS
A2780	Ovarian	wt	−	S/SS
A2780cis	Ovarian	wt	−	SS
A2780ADR	Ovarian	wt	−	Add/S

*Lung*				
NCI-H1770	NSCLC	R248W (hom.)	+++	SS
NCI-H1975	NSCLC	R273H (hom.)	+++	SS
NCI-H596	NSCLC	G245C (hom.)	+++	SS
NCI-H378	SCLC	Y163C (hom.)	+	SS
NCI-H1417	SCLC	175fs246* (hom.) (‘p53 null')	−	S/SS

Abbreviations: hom., homozygous; het., heterozygous; *, stop codon; fs, frame shift; ‘p53 null', no full-length p53; −, no p53 expression seen; +, weak p53 expression; +++, strong p53 expression; SCLC, small cell lung cancer; NSCLC, non-small cell lung cancer; Add, additive (CI=1.0±0.2); S, synergy (CI<0.8); SS, strong synergy (CI<0.5).

MTS, FMCA or Cell Titer-Glo assays were used for measurement of cell viability. CI was calculated using Additive model. It should be noted that the sequencing method used (Sanger sequencing and Single Strand Conformation Analysis) cannot distinguish between homozygous and hemizygous mutations. Also, ‘het.' refers to that both wt p53 and mut p53 are found in the sample. This can either be due to heterozygosity or to a presence of cells with different p53 status, which is not common in cancer cell lines but may occur in primary cancer cells

**Table 1b:** IC_50_ values and resistance factors for APR-246, platinum compounds and doxorubicin in the parental ovarian A2780 cell line and drug-resistant sublines

**Substance**	**A2780 (wt p53) IC**_**50**_ **(μM)**	**A2780cis (wt p53) IC**_**50**_ **(μM)**	**Resistance factor**	**A2780-CP20 (het. V172F p53) IC**_**50**_ **(μM)**	**Resistance factor**	**A2780ADR (wt p53) IC**_**50**_ **(μM)**	**Resistance factor**
Cisplatin	3.7±0.66	18±1.9***	4.8	40±4.6**	11	15±1.5**	4.2
Carboplatin	76±13	170±10***	2.2	425±42***	5.6	17±16**	2.3
Doxorubicin	0.12±0.047	0.31±0.054*	2.6	0.76±0.060***	6.4	2.1±0.59*	18
APR-246	23±1.6	20±1.1	0.83	37±2.3***	1.6	11±2.0***	0.48

FMCA was used for measurement of cell viability. *t*-test (two tailed, unpaired, unequal variance) was used for statistical analysis of differences in potency (IC_50_ values) of drugs in drug-resistant sublines compared with the parental cell line A2780; *P<0.05; **P<0.01; ***P<0.001. The results are mean±S.E.M. of at least three independent experiments

**Table 1c:** Results from combination studies with APR-246 and cisplatin in primary ovarian cancer cells

**Patient number**	**Histological description**	**p53 status**	**Combination APR-246 and cisplatin**
1	Serous adenocarcinoma, grade 2	P153H fs 180* (hom.) (‘p53 null')	SS
2	Serous adenocarcinoma, grade 3	C135A fs 169* (het.)	SS
3	Serous adenocarcinoma	Y220C (hom.)	SS
4	Poorly differentiated adenocarcinoma	wt p53	SS
5	Adenocarcinoma	Q165* (het.)	SS

Abbreviations: hom., homozygous; het., heterozygous; *, stop codon; fs, frame shift; ‘p53 null', no full-length p53; SS, strong synergy (CI<0.5).

FMCA assay was used for measurement of cell viability. CI was calculated using Additive model. It should be noted that the sequencing method used (Sanger sequencing and Single Strand Conformation Analysis) cannot distinguish between homozygous and hemizygous mutations. Also, ‘het.' refers to that both wt p53 and mut p53 are found in the sample. This can either be due to heterozygosity or to a presence of cells with different p53 status, which is not common in cancer cell lines but may occur in primary cancer cells

## Abbreviations

ABC: ATP-binding cassette
APR-246: 2-hydroxymethyl-2-methoxymethyl-1-azabicyclo [2,2,2] octan-3-one
BSO: buthionine sulfoximine
CI: combination index
GSH: glutathione
GSSG: glutathione disulfide
HGS: high-grade serous
i.v.: intravenous
MS: mass spectrum
MQ: 2-methylenequinuclidin-3-one
NSCLC: non-small cell lung cancer
PI: propidium iodine
ROS: reactive oxygen species
s.c.: subcutaneous
SCLC: small cell lung cancer
SSCA: single-strand conformation analysis
TrxR1: thioredoxin reductase 1
Wt: wild type
