# Supplementary material for: APR-246 overcomes resistance to cisplatin and doxorubicin in ovarian cancer cells
Source: Cell Death Dis. 2015 Jun 18;6(6):e1794–. doi: 10.1038/cddis.2015.143 (PMC4669826; doi:10.1038/cddis.2015.143)
Supplement: Supplementary Information [file cddis2015143x1.pdf]

Supplementary results

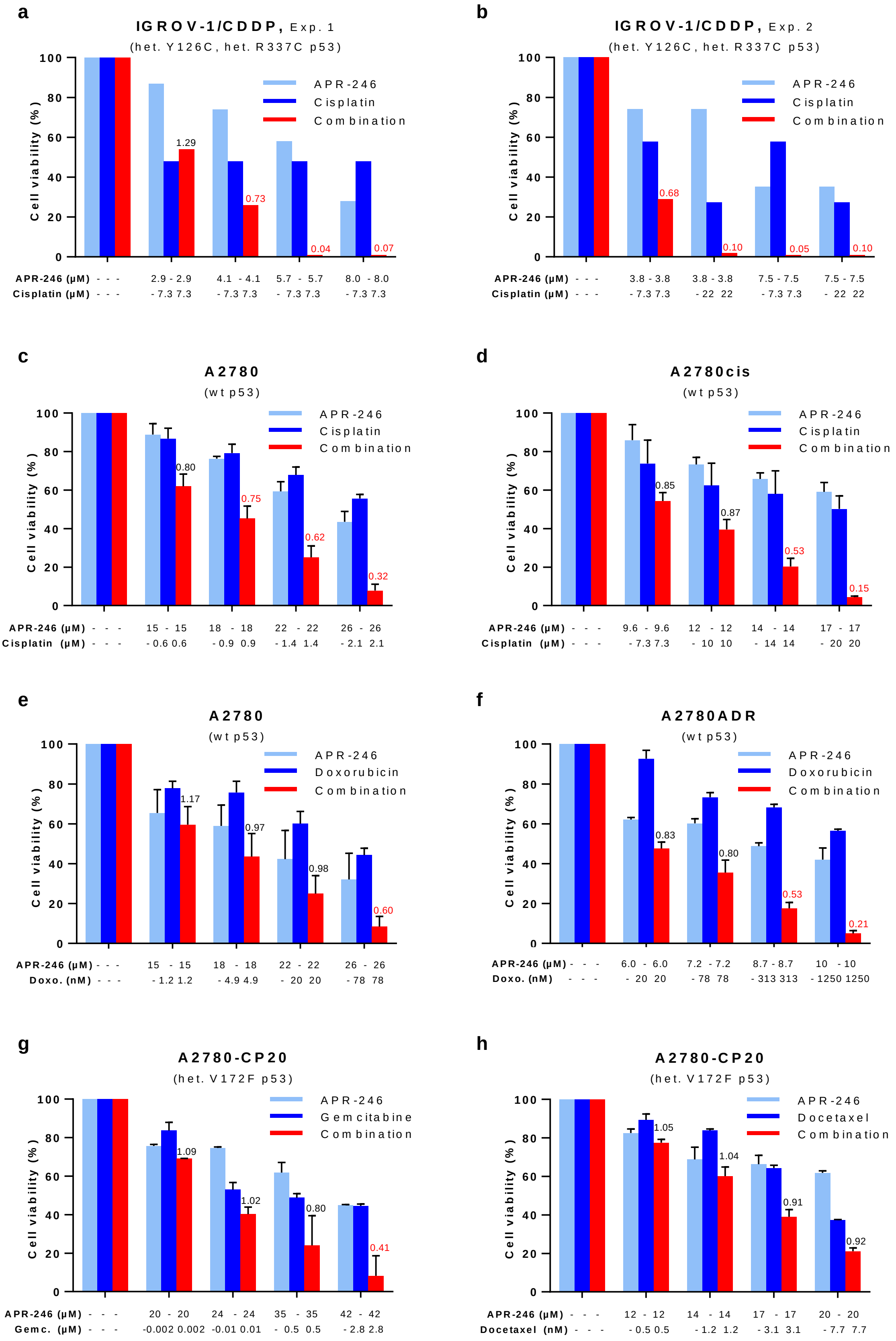

Supplementary Figure S1 Combination effects of APR-246 and cisplatin, doxorubicin, gemcitabine or docetaxel on cell viability of ovarian cancer cell lines with various p53 status and drug resistance. (**a** and **b**) APR-246 and cisplatin in cisplatin resistant IGROV-1/CDDP cells. (**c** and **d**) APR-246 and cisplatin in parental A2780 cells and cisplatin-resistant A2780cis cells. (**e** and **f**) APR-246 and doxorubicin in A2780 cells and the doxorubicin-resistant A2780ADR cells. (**g**) APR-246 and gemcitabine in cisplatin-resistant A2780-CP20 cells. (**h**) APR-246 and docetaxel in A2780-CP20 cells. The FMCA assay was used for measurement of cell viability. The results shown are mean±SEM (n≥2). For IGROV-1/CDDP two independent experiments are shown. Additive model was used for analysis of combination effects. Combination Index (CI) < 0.8 indicates synergistic, < 0.5 strong synergistic and < 0.3 outstanding synergistic effect. CI values < 0.8 are marked in red.

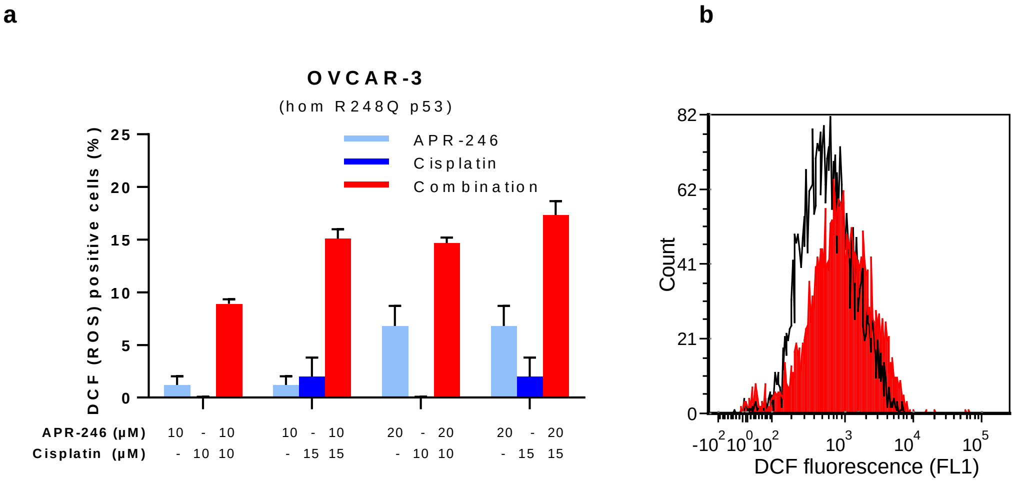

Supplementary Figure S2 Effect of APR-246 in combination with cisplatin on ROS levels in OVCAR-3 cells after 20 h treatment. ROS production was estimated by DCF staining and assessed by flow cytometry. (**a**) Factorial ANOVA indicated statistically significant synergistic interaction between cisplatin and APR-246 in the induction of ROS in OVCAR-3 cells ($p<0.01$). The results are shown as mean±SEM (n=2). (**b**) Flow cytometry overlay histogram showing the relative green DCF fluorescence (FL1). Untreated control is shown in black and combination (20 µM APR-246 and 10 µM cisplatin) in red. The histogram for the combination treatment is right shifted relative to the untreated control which indicates that ROS levels are increased after combination treatment with APR-246 and cisplatin.

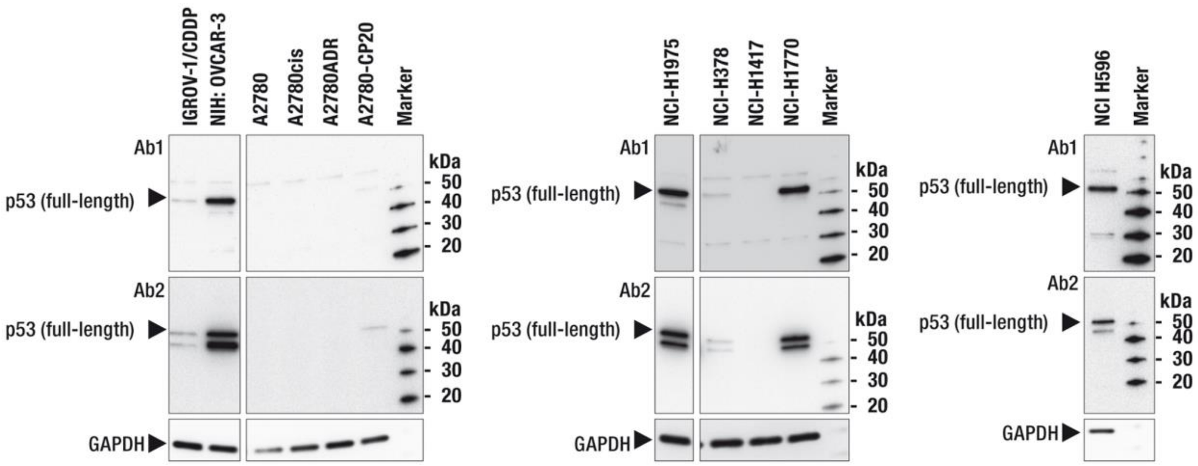

Supplementary Figure S3 p53 protein expression in ovarian and lung cancer cell lines. p53 expression was analysed by western blotting using a polyclonal anti-p53 antibody that binds strongly to the N-terminal of the p53 protein and more weakly to the DNA-binding region of p53 (#9282, Ab1), and a polyclonal anti-p53 antibody that also detects isoforms that are not full length (#FL-393, Ab2).

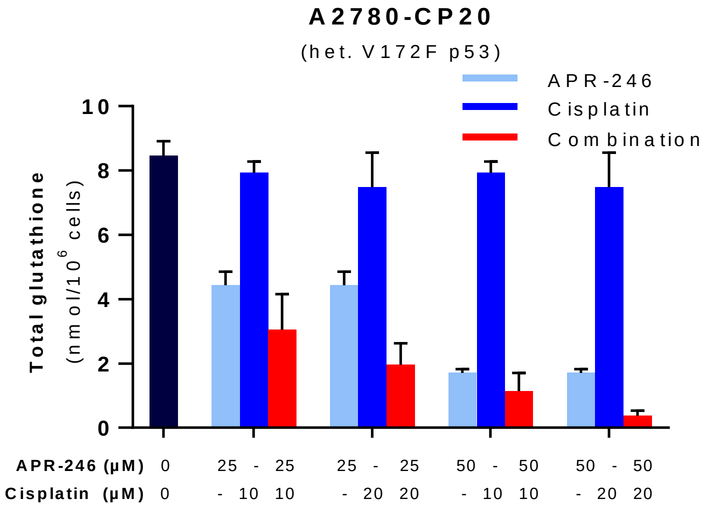

Supplementary Figure S4 Effect of APR-246, cisplatin and combination on glutathione levels in A2780-CP20 ovarian cancer cells. The total GSH levels (GSH + 2xGSSG) after treatment with APR-246, cisplatin and combination using Cayman glutathione assay kit are shown. The results are mean±SEM (n=2). The cell viability was > 85% for all samples.

Supplementary methods, additional information

Analysis of TP53 Gene Status

DNA was isolated using the Maxwell® 16 Cell DNA Purification Kit (Promega). PCR was performed on an Eppendorf thermocycler using HotStarTaq® Master Mix Kit (Qiagen). Exons 2-11 in the *TP53* gene were sequenced by Sanger sequencing. PCR amplification: The temperature protocol was as follows; initial denaturation and activation of the HotStar enzyme at 95°C for 15 min and then 35 cycles of denaturation at 95°C for 30 sec, annealing at 55°C for 30 sec and extension at 72°C for 45 sec. After the last cycle, 72°C for 5 min and then to 18°C. 3-5 µl of the PCR product was used for DNA sequencing with the use of the BigDye® Terminator v3.1 Cycle Sequencing Kit (AppliedBiosystems™). Data were analysed with the software ABI Sequencing Analysis v.5.4.

Single Strand Conformation Analysis: 1 µl of the PCR product was 32P-dATP labelled. The 32P-dATP labelling reaction consisted of 1 x Dream Taq buffer (2.0 mM $MgCl_2$), 0.25 U DreamTaq DNA polymerase (Fermentas), 200 µM dNTP, 0.5 mM of each primer and 10 µCi of 32P-dATP (PerkinElmer) in a 10 µl reaction volume. The DNA was initially denatured for 2 min at 95°C, and then denatured for 30 sec, annealed at 55°C for 30 sec and extended at 72°C for 30 sec for 12 cycles. Labelled PCR products were diluted 20-fold with 50% formamide/10 mM EDTA/0.1% SDS (containing xylene cyanol and bromophenol blue). After denaturation at 95°C for 5 min, the samples were immediately put on ice and loaded on to a native 6% polyacrylamide gel containing 10% glycerol. Electrophoretic separation of single strand DNA was performed at 10-12 W for 16-20 h. The gels were attached to filter paper and dried before exposure to x-ray film for 16-24 h. Mobility shifted bands, indicating a different secondary structure, were identified as mutated bands, excised, and DNA was eluted and used in a re-amplification PCR reaction for direct DNA sequencing according to the Sanger sequencing protocol.

Primers used for PCR amplification, Single Strand Conformation Analysis (SSCA), and Sanger sequencing:

5´>3´	
Exon 2-3	GGAGGGGGTTCCTTCTCTG
	AAAAGAGCAGTCAGAGGACCAG
Exon 4	CTGAGGACCTGGTCCTCTGAC
	ATACGGCCAGGCATTGAAGT
Exon 5+6	TTCACTTGTGCCCTGACTT
	CGGAGGGCCACTGACAACCA
Exon 5A	TTCAACTCTGTCTCCTTCCT (SSCA)
	ACTGCTTGTAGATGGCCATG (SSCA)
Exon 5B	GTGCAGCTGTGGGTTGATTC (SSCA)
	CAGCCCTGTCGTCTCTCCAG (SSCA)
Exon 6	TTGCCCAGGGTCCCCAGGCC (SSCA)
	AACCCCTCCTCCCAGAGAC (SSCA)
Exon 7	CCTCCCCTGCTTGCCACAGG
	GGAAGAAATCGGTAAGAGGTGG
Exon 8	CTGCCTCTTGCTTCTCTTTT
	CTCCTCCACCGCTTCTTGTC
Exon 9	GCCTCAGATTCACTTTTATCACC
	CTGGAAACTTTCCACTTGAT
Exon 10	CTTGAACCATCTTTTAACTCAGG
	AATCCTATGGCTTTCCAACCTAGG
Exon 11	CTTCAAAGCATTGGTCAGGG
	GGGTTCAAAGACCCAAAACC

Western Blotting

2x RIPA buffer: 0.87766 g NaCl, 1 ml 100x Triton, 0.5 ml 20% SDS, 5 ml 1 M Tris pH 8.0 + H_2O up to 50 ml. Total protein concentration was determined with the BCA-method as described in.[1] Equal amount of protein from all samples was added to 2x Laemmli buffer (Bio-Rad) supplemented with 2-mercaptoethanol (Bio-Rad, 1:20 in the Laemmli buffer), and heated to 95°C for 5 min before separated by electrophoresis on a 4-15% TGX-gel (Bio-Rad) and transferred to PVDF membrane using a semi-dry trans blot system (Bio-Rad). The membranes were blocked in Tris-buffered saline containing 5% w/v non-fat dry milk and 0.1% Tween-20, followed by incubation with primary antibody overnight at 4°C, and with secondary antibody for 1 h at room temperature. Antibodies were diluted in Tris-buffered saline (TBS) containing 5% w/v non-fat dry milk and 0.1% Tween-20. The proteins were visualized by enhanced chemiluminescence system (ECL prime, VWR) and detected using a CCD camera (LAS1000 Fujifilm Tokyo Japan).

Cell Lines and Cell Culture

Supplementary Table S1 Cancer cell lines.

Tumor type	Cell line	Source	Morphology	Disease	Derived from	Growth media	Previously reported p53 status[1]
Ovarian	A2780	93112519, Sigma	Epithelial	adenocarcinoma	tumor tissue	#	wt [2]
Ovarian	A2780cis	93112517, Sigma	Epithelial	adenocarcinoma	tumor tissue	#¤	K351N [3] ^
Ovarian	A2780-CP20	Fox Chase Cancer Center, Philadelphia (Dr. StephenWilliams)	Epithelial	adenocarcinoma	tumor tissue	#	V172F [4]
Ovarian	A2780ADR	93112520, Sigma	Epithelial	adenocarcinoma	tumor tissue	#¤¤	
Ovarian	NIH:OVCAR-3	HTB-161, ATCC	Epithelial	adenocarcinoma	ascites	# + 1 % insulin, Transferrin and selenium A supplement, or in RPMI 1640 + 10 mM Hepes + 1 mM NaPyr, 2.5 g/L glucose, 0.01 mg/ml bovine insulin and 20 % FBS	R248Q [5]
Ovarian	IGROV-1/ CDDP	Pharmacell	Epithelial-like	adenocarcinoma	tumor tissue	RPMI 1640 + 10 mM Hepes+ 1 mM NaPyr, 2.5 g/L glucose, 0.01 mg/ml bovine insulin and 20 % FBS	Y126C [2] ^^
NSCLC	NCI-H1770	CRL-5893, ATCC		carcinoma	metastases (lymph node)	#	R248W N247N [2]
NSCLC	NCI-H1975	CRL-5908, ATCC	Epithelial	adenocarcinoma	tumor tissue	#	R273H [2]
NSCLC	NCI-H596	HTB-178, ATCC	Epithelial	adenosquamous carcinoma	chest wall	#	G245C [6]
SCLC	NCI-H1417	CRL-5869, ATCC		carcinoma	tumor tissue	#	R175fs246* [7]
SCLC	NCI-H378	CRL-5808, ATCC	Epithelial	carcinoma	pleural effusion	#	Y163C [8]

[1] For the present publication's sequencing results of all cell lines, see Table 1a.

[2-8] Refers to supplementary references.

^The A2780cis cells had been reported to carry a K351N mutation, but the cell line has wt p53 according to our sequencing.

^^For the IGROV-1/CDDP cell line we found two mutations of which the R337C (het.) mutation had not been previously reported.

wt = wild type

het. = heterozygous

fs = frame shift mutation

* = stop codon

NSCLC = non small cell lung cancer

SCLC = small cell lung cancer

RPMI 1640 + 10 % FBS + 2 mM glutamine

¤ To retain cisplatin resistance in A2780cis, 1 µM cisplatin was added to the culture media every 3 passages.

¤¤ To retain doxorubicin resistance in A2780ADR, 0.1 µM doxorubicin was added to the culture media at least once a month.

The A2780cis and A2780ADR cells were drug free for at least 48 h before they were used in FMCA assay. The A2780-CP20, NCI-H1770, NCI-H1975, NCI-H1417 and NCI-H378 cell lines were authenticated by short tandem repeat (STR) analysis (IGROV-1/CDDP was not described in ATCC or DSMZ databases for cell authentification). All other cell lines were obtained directly from cell banks and passaged for fewer than 6 months after their receipt, and reauthentication was not needed.

Cell Viability Assays

The fluorometric microculture cytotoxicity (FMCA) assay: 96-well plates with v-shaped wells (Nunc) were prepared with test substances at 10x the desired concentration. The prepared substance plates were kept at -80°C until they were used. The cell suspension was seeded out with $1.5*10^4$ cells/ml (3000 cells/well) - $6.0*10^4$ cells/ml (12000 cells/well) in thawed substance plates and incubated for 72 h in a humidified atmosphere, at 37°C and with 5% CO_2. The cell plates were washed and incubated for another 40 min with 100 µl of 1 µl fluorescein diacetate (FDA) (Sigma)/ml physiological buffer (Q2) per well.[9] The Q2 buffer was kept in room temperature and before use the amount of needed buffer was stored approximately for 15-20 min at 37°C. The fluorescence was measured at 538 nm in a fluorometer (Fluoroscan II absorbance reader (Labsystems Oy), FluoStar Optima (BMG Labtech) or BMG Fluo Omega (BMG Labtech)). The fluorescence is proportional to the number of cells with intact plasma membrane. FMCA measures the esterase activity of cells with intact plasma membranes by measuring fluorescence generated when the nonfluorescent probe, FDA, is hydrolysed to fluorescein.

WST-1 assay: Cells were seeded at 3000 cells/well, 100 µl/well, in 96-well plates with flat bottom (Costar, Corning Incorporated). Following 24 h incubation at 37°C, 5% CO_2, substance containing culture medium was added, 100 µl/well, giving a total volume of 200 µl/well. The plates were incubated for 72 h at 37°C, 5% CO_2. Thereafter, WST-1 reagent (Roche) was added to all wells (final dilution 1:10) and the plates were incubated for 60 minutes at 37°C, 5% CO_2 before the absorbance was measured in a microplate absorbance reader (Multiskan EX photometric microplate absorbance reader, Thermo). WST-1 cell viability assay is based on the cleavage of the tetrazolium salt WST-1 (4-[3-(4-iodophenyl)-2-(4-nitrophenyl)-2H-5-tetrazolio]-1,3-benzene disulfonate). In metabolically active cells WST is cleaved by mitochondrial dehydrogenases, whereby a water-soluble formazan dye is produced. The amount of formazan dye is directly proportional to the number of metabolically active cells.

Cell Titer-Glo® assay: Cells were seeded in 96-well flat bottomed black microplates and incubated at 37°C, 5% CO_2, for 24 h before treatment. Adherent cell lines were seeded at 3000 cells/well and suspension cell lines at 6000 cells/well, 150 µl/well. Substances were added. After 72 h incubation in CO_2 incubator, cells were lysed using 50 µl/well of Cell Titer-Glo reagent solution (Promega). After

mixing for 10 minutes, plates were read in a luminometer (Envision - PerkinElmer). Cell Titer-Glo is a luciferase based assay that evaluates the number of metabolically active cells by quantification of ATP.

MTS assay: Cells were seeded in 96-well flat bottom plates (Nunc) and incubated at 37°C, 5% CO_2, in RPMI 1640 medium supplemented with 10% FBS for 24 h before treatment. Seeding density was adapted for each cell line based on doubling time, 3000 cells/well - 12000 cells/well, 190 µl/well. Cell lines were incubated for 72 or 96 h (depending on cell line) with test substances in a total volume of 200 µl. MTS (#G1111, Promega) and PMS (#P9625, Sigma) detection reagents were mixed, in a ratio of 20:1, thereafter 40 µl of the combined solution were added to each well. Culture plates were incubated for 2 or 4 h at 37°C. Absorbance (OD) was measured at 490 nm using VICTOR^{3TM} 1420 multilabel counter (Wallac, PerkinElmer, Courtaboeuf, France). MTS assay is based on that the tetrazolium compound, MTS (3-(4.5-dimethylthiazol-2-yl)-5-(3-carboxymethoxyphenyl)-2-(4-sulfophenyl)-2H-tetrazolium), a yellow tetrazole, is reduced by an electron coupling reagent, PMS (phenazine methosulfate), to a purple formazan in living cells.

Analysis of Combination Results Using Additive Model

In samples with two co-incubated substances, a predicted cell viability (decimal form, i.e., % x 0.01) was calculated according to the following formula: Predicted cell viability (decimal form) = cell viability of substance 1 (decimal form) x cell viability of substance 2 (decimal form). A “combination index” (CI) was then calculated as the measured cell viability of the sample with two co-incubated substances divided by the predicted cell viability: CI = measured cell viability / predicted cell viability. CI = 1±0.2 indicates additive effect, < 0.8 indicates synergistic and < 0.5 strong synergistic effect. CI > 1.2 indicates sub-additive or antagonistic effects.

In Vivo Xenograft Efficacy Study

When tumors had reached a volume of 90-170 mm^3, the mice were divided into four groups of 10 mice, and randomly assigned to treatment and control groups. Tumor volumes were calculated with the formula: Tumor vol = length x width2 x 0.5. The tumor growth inhibition (% TGI) was calculated with the formula % TGI = (((Tumor volume control group Day 8 - Tumor volume control group Day 1) -

(Tumor volume treatment group Day 8 - Tumor vol treatment group Day 1)) / (Tumor volume control group Day 8 - Tumor volume control group Day 1)) x 100.

Evaluation of Active Caspase-3 in Tumors

The excised tumors were fixed for 24 h in 10% buffered formalin at room temperature and embedded in paraffin. Serial sections (2-4 µm thickness) were cut and stained with haematoxylin and eosin (H&E) for histopathology. For immunohistochemistry, sections were deparaffinised in xylene, hydrated in graded ethanol and tap water and transferred to PBS. Tumor sections were subjected to heat-induced epitope retrieval in citrate buffer pH 6.0 (Vector) by a pressure cooker for 3 min at 120°C and then cooled for 30 min. Endogenous peroxidase was blocked incubating the slices with 3% hydrogen peroxide in PBS for 10 min. After blocking the tissues were washed two times with PBS containing 0.05% Tween-20 (PBST) and incubated for 30 min with 10% normal goat serum (Vector) in PBST (blocking solution). Blocking solution was substituted by primary monoclonal rabbit anti-active Caspase-3 antibody (Cell Signaling) and incubated for 1 h at room temperature. Tumors were then rinsed twice with PBST and incubated for 30 min at room temperature with the DAKO EnVision + Rabbit Polymer System. After washing with PBST, sections were incubated for 1-3 min with liquid DAB + substrate chromogen system (3,3 diaminobenzidine, Dako). They were then washed three times with tap water, counterstained for 3 minutes with Gill's hematoxylin (Bio Optica), washed with tap water, dehydrated in graded ethanol/xylene and mounted.

Glutathione Assay

The Cayman kit contains GS reductase, which generates glutathione (GSH) from glutathione disulfide GSSG. Then GSH reacts with DTNB (5,5'-dithio-bis-2-(nitrobenzoic acid), Ellman's reagent), producing a yellow coloured 5-thio-2-nitrobenzoic acid (TNB), which is measured by absorbance. The rate of formation of TNB is proportional to the GSH concentration in the sample. Since GS reductase is present in the kit, the sum of glutathione and glutathione disulfide is detected. When cell diameters were measured using a cell Coulter counter the approximate observed cell volumes were 1.56 pl for A2780 and 1.55 pl for A2780-CP20, and accordingly the total GSH (GSH + 2xGSSG) mM concentrations were ~0.64 times the displayed nmol/10^6 cells concentrations.

References

1. Smith PK, Krohn RI, Hermanson GT, Mallia AK, Gartner FH, Provenzano MD*, et al.* Measurement of protein using bicinchoninic acid. *Anal Biochem* 1985, **150**(1)**:** 76-85.

2. Forbes S, Clements J, Dawson E, Bamford S, Webb T, Dogan A*, et al.* Cosmic 2005. *British journal of cancer* 2006, **94**(2)**:** 318-322.

3. Muscolini M, Montagni E, Caristi S, Nomura T, Kamada R, Di Agostino S*, et al.* Characterization of a new cancer-associated mutant of p53 with a missense mutation (K351N) in the tetramerization domain. *Cell Cycle* 2009, **8**(20)**:** 3396-3405.

4. Skilling JS, Squatrito RC, Connor JP, Niemann T, Buller RE. p53 gene mutation analysis and antisense-mediated growth inhibition of human ovarian carcinoma cell lines. *Gynecol Oncol* 1996, **60**(1)**:** 72-80.

5. Yaginuma Y, Westphal H. Abnormal structure and expression of the p53 gene in human ovarian carcinoma cell lines. *Cancer research* 1992, **52**(15)**:** 4196-4199.

6. Mitsudomi T, Steinberg SM, Nau MM, Carbone D, D'Amico D, Bodner S*, et al.* p53 gene mutations in non-small-cell lung cancer cell lines and their correlation with the presence of ras mutations and clinical features. *Oncogene* 1992, **7**(1)**:** 171-180.

7. Phelps RM, Johnson BE, Ihde DC, Gazdar AF, Carbone DP, McClintock PR*, et al.* NCI-Navy Medical Oncology Branch cell line data base. *J Cell Biochem Suppl* 1996, **24:** 32-91.

8. Takahashi T, Nau MM, Chiba I, Birrer MJ, Rosenberg RK, Vinocour M*, et al.* p53: a frequent target for genetic abnormalities in lung cancer. *Science* 1989, **246**(4929)**:** 491-494.

9. Lindhagen E, Nygren P, Larsson R. The fluorometric microculture cytotoxicity assay. *Nat Protoc* 2008, **3**(8)**:** 1364-1369.
